# ﻿Utility of cytochrome c oxidase I for the deciphering of unstable phylogeny and taxonomy of gorals, genus *Nemorhaedus* Hamilton Smith, 1827 (Bovidae, Ovibovina)

**DOI:** 10.3897/zookeys.1181.108019

**Published:** 2023-10-02

**Authors:** Petr Hrabina, Ludmila Pernerová, Josef Suchomel, Jan Robovský

**Affiliations:** 1 Department of Zoology, Fisheries, Hydrobiology and Apiculture, Mendel University in Brno, Zemědělská 1, Brno, 61300, Czech Republic Mendel University in Brno Brno Czech Republic; 2 Department of Zoology, Faculty of Science, University of South Bohemia, Branišovská 1760, České Budějovice, 37005, Czech Republic University of South Bohemia České Budějovice Czech Republic; 3 Liberec Zoo, Lidové sady 425/1, Liberec, 46001, Czech Republic Liberec Zoo Liberec Czech Republic

**Keywords:** COI, mitochondrion, *Naemorhedus*, voucher specimen

## Abstract

Gorals represent ungulate mammals of the Palearctic and Indo-Malayan realms that face habitat destruction and intense hunting pressure. Their classification has been the subject of various (mainly genetic) assessments in the last decade, but some results are conflicting, hampering some conservation-based decisions. Genetic sampling of gorals has increased considerably in recent years, at least for mitochondrial (mt) DNA. Results based on two mt genes (cytochrome *b* and the D-loop) are currently available. Still, the utility of cytochrome oxidase subunit I remains unanalysed, even though it belongs among the gene markers that enable a correct species identification in mammals. This study examines phylogenetic relationships and species delimitation in gorals using all currently available cytochrome oxidase subunit I sequences, including the not yet analysed goral population from Pakistan. Our results of various phylogenetic approaches, such as maximum parsimony, likelihood and Bayesian inference, and exploration of species boundaries via species delimitation support the validity of six species of goral, namely *N.baileyi*, *N.caudatus*, *N.cranbrooki*, *N.evansi*, *N.goral*, and *N.griseus*. This result accords well with results based on other mt genes, especially the cytochrome *b* from the highly exhaustive data sampling. Our study also summarises common sources of errors in the assessment of goral phylogeny and taxonomy and highlights future priorities in understanding goral diversification.

## ﻿Introduction

Gorals are elusive herbivore mammals that occupy the mixed feeder niche in the Palearctic and Indo-Malayan realms (Isarankura Na Ayudhya et al. 2022). The genus occupies a vast distribution range across Asia, spanning several biogeographical zones and biomes (Damm and Franco 2014; Dinerstein et al. 2017); however, they seem to be susceptible to natural climatic and other environmental changes and the negative effects of human activities (Suraprasit et al. 2020a; Isarankura Na Ayudhya et al. 2022). Their sensitivity is associated with habitat preferences (close ties to steep cliffs and rocky crags) and behavioural constraints. As a resident species with a high degree of fidelity to small territories in both sexes (Cho et al. 2014, 2016; Teng et al. 2021), they are highly vulnerable to severe weather conditions (abnormal snow depth), natural predators, intense habitat destruction and heavy hunting pressure (Mishra et al. 1998; Rabinowitz 1999; Aiyadurai et al. 2010; Perveen et al. 2013; Velho and Laurance 2013; Shakeel et al. 2015; Park and Hong 2021; Shepherd et al. 2022). Gorals are subjects of the illegal meat and fur trade, their body parts are used in traditional medicine, and they are considered trophies (Mishra et al. 2006; Shepherd et al. 2022). However, other factors, such as disease, e.g., goatpox, whose high emergence has recently been reported in Northeast India (Bora et al. 2021), may also be important in causing declines. In effect, all species are currently facing declines and further isolation in populations (Lee and Rhim 2002; Duckworth and MacKinnon 2008; Abbas et al. 2012; Saeed et al. 2012; Bragina et al. 2020; Nijhawan 2020). Their sedentary life might deepen the differentiation of populations over time and lead to a reduction of effective population sizes in fragmented habitats, which could be reliably detected by mitochondrial DNA (mtDNA) (Brown et al. 2004; Roberts 2006; Grandi et al. 2018).

Gorals have already been the subject of some conservation-based population genetics studies (Min et al. 2004; Yang et al. 2019). Most genetic studies of gorals, however, have been focused mainly on alpha taxonomy, which is crucial in establishing future effective conservation management. The recent (mainly genetic) assessments are based solely on mtDNA (e.g., Mori et al. 2019; Li et al. 2020; Joshi et al. 2022). Although mtDNA has some known limitations (Wan et al. 2004; Fišer Pečnikar and Buzan 2014; Ritchie et al. 2016; Norman et al. 2019; Kowalczyk et al. 2021), these surveys are often logical in respect of available comparative gene and taxon sampling and availability from degraded samples, in comparison to nuclear or genomic DNA (Wan et al. 2004; Wandeler et al. 2007; Beja-Pereira et al. 2009). Therefore, various genetic assessments of different organisms still utilize mtDNA (Tobe et al. 2010; Gutiérrez et al. 2017; Kowalczyk et al. 2021; Leigh et al. 2021). Gorals themselves have been analysed using cytochrome *b* and the D-loop (Joshi et al. 2022), as well as the complete mitochondrial genome (Hassanin et al. 2012; Mori et al. 2019; Li et al. 2020). In contrast, the utility of cytochrome oxidase subunit I (COI) remains unanalysed, even though it belongs to the gene markers that enable a correct species identification in many animal groups, including mammals (Tobe et al. 2010). COI was selected as the optimal marker for barcoding (e.g., within the International Barcode of Life initiative (https://ibol.org/about/dna-barcoding/) and in Tobe et al. 2009, 2010; and Fišer Pečnikar and Buzan 2014). The current coverage of goral taxa for COI resembles the coverage for other mt genes, cyt *b* and the D-loop (for details, see Suppl. material 1), but it also contains specimens from western limits of goral distribution, specifically from Pakistan (Machiara National Park), that have not yet been inspected for genetic data. These specimens can be labelled as *Nemorhaedusbedfordi* (sensu Groves and Grubb 2011) or *N.goral* (sensu Hrabina 2015), which partly demonstrates that the current taxonomy of gorals is unstable, conflicting, and somewhat confusing.

It is worth noting that attempts to decipher goral phylogeny and species diversity are hampered by several complicated factors, as follows: relative phenotypic similarity of goral taxa; rarity of localised collection specimens (Adlerberg 1932; Dolan 1963; Groves and Grubb 2011; PH – pers. obs.); and chromosomal conservatism (Soma et al. 1980, 1987). These parameters might be responsible for a higher probability of misidentification of goral voucher specimens, which appears to be a major cause of error in efforts to revise goral taxonomy. Concerning misidentifications, some studies (Li et al. 2020; Joshi et al. 2022) have already revealed a misidentification based on the geographic origin of samples and their genetic affinities to other localised samples (Table 1).

**Table 1.** Detailed information for all goral sequence included in this study. Species are arranged alphabetically by scientific name.

Species name	Accession Numbers	Provenance	marker available	Reference
*N.baileyi*	JN632663	Yigong village, Linzhi City, Tibet, China	complete mitochondrion	Hassanin et al. 2012
*N.baileyi*	KP203894	Yigong village, Linzhi City, Tibet, China	complete mitochondrion	Shi et al. 2016
*N.caudatus*	FJ469673	South Korea	complete mitochondrion	Jang and Hwang 2010
*N.cranbrooki*	MN853098	Awadam village, Putao District, Myanmar	complete mitochondrion	Li et al. 2020
*N.cranbrooki*	MN853099	Tala Htu village, Putao District, Myanmar	complete mitochondrion	Li et al. 2020
*N.cranbrooki*	MN853101	Putao District, Myanmar	complete mitochondrion	Li et al. 2020
*N.cranbrooki*	MN853102	Ziyadam village, Putao District, Myanmar	complete mitochondrion	Li et al. 2020
*N.cranbrooki*	MN853103	Putao District, Myanmar	complete mitochondrion	Li et al. 2020
*N.evansi*	JN632664	Chiang Mai Province, Thailand	complete mitochondrion	Hassanin et al. 2012
*N.evansi*	MF155891	Fanjingshan National Nature Reserve, Guizhou Province, China	complete mitochondrion	Liu and Zhang 2018
*N.evansi*	MN853096	Putao District, Myanmar	complete mitochondrion	Li et al. 2020
*N.goral*	OK244620	Machiara National Park, Azad Jammu and Kashmir, Pakistan	complete COI gene	A. Naseem (The University of Sargodha, Pakistan) direct submission to GenBank
*N.goral*	OK244621	Machiara National Park, Azad Jammu and Kashmir, Pakistan	complete COI gene	A. Naseem (The University of Sargodha, Pakistan) direct submission to GenBank
*N.goral*	OK244622	Machiara National Park, Azad Jammu and Kashmir, Pakistan	complete COI gene	A. Naseem (The University of Sargodha, Pakistan) direct submission to GenBank
*N.griseus*	FJ207532	Central China	complete mitochondrion	Hassanin et al. 2009
*N.griseus*	JX188255	China	complete mitochondrion	Yang et al. 2013
*N.griseus*	KF500173	Wufeng Tujia Autonomous County, Hubei Province, China	complete mitochondrion	Zhang et al. 2015
*N.griseus*	KT878720	Tangjiahe National Nature Reserve, Sichuan Province, China	complete mitochondrion	Liu and Jiang 2017
*N.griseus*	MG591488	Wutai County, Shanxi Province, China	complete mitochondrion	Ren et al. 2018
*N.griseus*	MG865962	western Sichuan Province, China	complete mitochondrion	Yao et al. 2019

Concerning an imbalance between morphological and genetic revisions of gorals, although some authors have tried to compare taxa using morphological data (Suppl. material 1), only Dolan (1963) and Groves and Grubb (2011), possibly Groves and Grubb (1985), and Grubb (2005) have revised goral taxonomy using standard comparative methods (Mayr 1969; Mayr and Ashlock 1991; Groves and Grubb 2011). Unfortunately, the detailed arguments and datasets behind taxonomic opinions in the case of Groves and Grubb (1985), Grubb (2005), and somewhat in Groves and Grubb (2011), remain unspecified. Genetic studies are also confounded by the above complicating factors, as four taxonomic studies have appeared in the last decade with markedly different results (Hassanin et al. 2012; Mori et al. 2019; Li et al. 2020; Joshi et al. 2022).

Although some different phylogenetic relationships and affinities revealed by genetic or morphological studies seem to be contradictory, the contradictions arise only from taxa sampling and voucher (mis)identifications (Suppl. material 1; Table 2). After the abandonment of some extremes, such as the oversplitting of variability (e.g., 15 new goral species described by Pierre-Marie Heude; see Heude 1894; Braun et al. 2001; Grubb 2005) or the probable overlumping (Haltenorth 1963, into only one goral species), morphology-based taxonomic revisions or reviews have converged to form six valid taxa of gorals since 2005. The molecular assessment of gorals has argued for three to seven valid taxa within three to six species of gorals (Suppl. material 1).

**Table 2.** List of goral complete mitochondrial genomes revised by different sources.

Accession Numbers	Organism (as specified in GenBank)	Li et al. (2020)	Joshi et al. (2022)	the present study	Data used for a revision
JN632664	*Nemorhaedusgriseus*	*Nemorhaedusevansi*	*Nemorhaedusevansi*	*Nemorhaedusevansi*	GD, PR, SGO
MF155891	*Nemorhaedusgriseus*	*Nemorhaedusevansi*	*Nemorhaedusevansi*	*Nemorhaedusevansi*	GD, PR
MG865962	*Nemorhaedusgoral*		*Nemorhaedusgriseus*	*Nemorhaedusgriseus*	GD, PR, SGO
MG591488	*Nemorhaedusgoral*		*Nemorhaedusgriseus*	*Nemorhaedusgriseus*	GD, PR, SGO
KT878720	*Nemorhaedusgoral*		*Nemorhaedusgriseus*	*Nemorhaedusgriseus*	GD, PR, SGO
JX188255	*Nemorhaedusgoral*			*Nemorhaedusgriseus*	GD, PR
MN853096	*Nemorhaedusevansi*			hybrid between *N.cranbrooki* and *N.evansi*?	PF

Abbreviations: GD – genetic distances, PF – phenotypic features, PR – phylogenetic relationships, SGO – specific geographic origin.

Considering all noted factors, especially the limited sampling and unstable phylogeny and taxonomy of gorals, they deserve further assessment, since their concise taxonomy might be helpful for further conservation actions in respect of international and national laws. The current study aims to inspect the utility of COI for both the detection of phylogenetic relationships among goral taxa and the inspection of diversification among available goralCOI voucher specimens, using genetic distances and single-locus species delimitation.

Gorals seem to represent an ideal model for these aims among caprines due to the available taxon-population sampling (i.e., due to the highest available numbers of complete mitogenomes), their sedentary lifestyle, the philopatry of females (Heptner et al. 1988; Yang et al. 2019) and perhaps their well-developed reproductive isolation mechanism, expressed as hybrid infertility (Volf 1976). Since gorals and serows represent the sister group to arctic dwelling musk-oxen (Hassanin et al. 2009), a much heavier and cold-adapted species, only one of which has survived, gorals might offer insight into the fascinating diversification and evolution of subtribe Ovibovina and all caprines (tribe Caprini).

## ﻿Materials and methods

Originally, we used 134 complete COI sequences (altogether 1545 bp) of gorals and other members of the tribe Caprini (according to Hassanin et al. 2012 and Groves and Grubb 2011) using the GenBank database (available in November 2022; for GenBank accession numbers see Suppl. material 2). If a complete COI sequence was not available by itself, it was extracted from the complete mitochondrial genomes. Various phylogenetic analyses (see below) of this dataset with 134 taxa recovered an efficient resolution of COI for separating genera and species, but it was not able to provide a robust phylogenetic relationships among caprine genera. Similar observation was made by Tobe et al. (2010), who identified cyt *b* as the better marker for reconstructing mammalian phylogenies, compared to COI. According to these results, and general compatibilities with results by Hassanin et al. (2009, 2012) based on complete mitogenomes, we restricted the dataset to only goral sequences (Table 1) with a serows (*Capricornis* sp.) as the outgroup. Since the spelling of the generic name of ‘goral’ is questionable (see Groves and Grubb 2011), we searched for both variants in the GenBank database, i.e., *Nemorhaedus* and *Naemorhedus*, although the NCBI Taxonomy Browser recognized it as *Naemorhedus* within the subfamily Caprinae in the family Bovidae.

Considering similarities among goral species and some taxonomic inertia in this group during recent decades, all sequences were checked according to their localities using taxonomically sensitive sources (Groves and Grubb 2011; Hrabina 2015; Castelló 2016). This approach is sometimes essential for the correct interpretation of results (Ouellette 2001; Müller et al. 2013; Steppan and Schenk 2017; Bryja et al. 2018; Li et al. 2018). Additionally, our revision of taxa specification was made based on previously revealed misidentifications (e.g., Li et al. 2020; and Joshi et al. 2022) or on our examination of monophyly and genetic distances of sequences to other localised sequences.

The sequences were aligned using the MAFFT version 6 automatic multiple alignment programme for amino acid or nucleotide sequences (Katoh et al. 2009). They were subsequently adjusted manually using Bioedit (Hall 1999). Prior to the analyses, COI sequences were translated into amino acids in MEGA11 (Tamura et al. 2021) using the vertebrate mitochondrial translation code. This procedure did not detect any stop codons or gaps, suggesting that all protein-coding sequences were functional, and no pseudogenes were amplified.

Genetic distances were estimated based on Kimura’s two-parameter model (K2P) of substitutions in MEGA5 (Tamura et al. 2011). We also estimated uncorrected genetic distances in the same software. This model has been selected for comparisons of other published distances for mammals (Bradley and Baker 2001; Baker and Bradley 2006).

The dataset was examined using various approaches: neighbour-joining (**NJ**), maximum parsimony (**MP**), maximum likelihood (**ML**) and Bayesian inference methods (**BI**), respectively, within MEGA11 (Tamura et al. 2021) and MrBayes version 3.1.2 (Huelsenbeck and Ronquist 2001; Ronquist and Huelsenbeck 2003). The best‐fitting substitution model for DNA sequence evolution was selected by MEGA11 (Tamura et al. 2021) under the Bayesian information criterion and Akaike information criterion. This evolution model, HKY+G+I, was determined for the complete sequence of COI. Bayesian phylogenetic analysis was performed with a Metropolis‐coupled Markov chain Monte Carlo algorithm (Altekar et al. 2004) within MrBayes version 3.1.2 (Huelsenbeck and Ronquist 2001; Ronquist and Huelsenbeck 2003). Nucleotide data were run for 5,000,000 generations with two runs, four chains for each run, and a sampling frequency of every 100^th^ generation; we discarded the first 10% of trees as burn-in. Convergence of MCMC analyses was diagnosed by the average standard deviation of split frequencies (**ASDSF**) within MrBayes version 3.1.2 (for references see above) and the effective sample size (**ESS**) value of the trace within Tracer v1.7.1 (Rambaut et al. 2018).

The observed ASDSF values of around 0.002–0.003 and effective sample sizes of MCCM per each run of at least around 1107 meet the criteria for recommended convergence thresholds according to Lakner et al. (2008) and Fabreti and Höhna (2021).

To assess nodal support, the bootstrapping with 250 replicates (i.e., within the recommended range by Pattengale et al. 2010) for the NJ, MP and ML analyses and the posterior probabilities for the BI analysis were used and implemented in MEGA11 (Tamura et al. 2021) and MrBayes version 3.1.2 (Huelsenbeck and Ronquist 2001; Ronquist and Huelsenbeck 2003), respectively. We considered bootstrap values ≥ 75% and posterior probability values ≥ 0.95 as strong support, respectively.

To explore species boundaries in the COIML and BI trees, we calculated several statistics using the Species Delimitation plugin (Masters et al. 2011) by Geneious, version 10.0.5 (Kearse et al. 2012). This plugin allows users to assign terminals of the detected phylogenetic tree to putative species, which we found using the most sensitive taxonomies of gorals. The Species Delimitation Plugin calculates several statistics relating to the phylogenetic exclusivity of each putative species, the probabilities that such exclusivity has arisen by chance in a random coalescent process, and the degree to which the species can be diagnosed (Masters et al. 2011; Gutiérrez et al. 2017). The plugin reports the following statistics (Masters et al. 2011): Intra Dist, the average pairwise tree distances among members of the putative species; Inter Dist, the average pairwise tree distance between the members of one putative species and the members of the closest second putative species; Intra/Inter, the ratio of Intra Dist to Inter Dist; P ID (Strict), the mean probability of correctly identifying an unknown member of the putative species using the criterion that it must fall within but not be sister to the species clade in a tree; P ID (Liberal), the mean probability of correctly identifying an unknown member of the putative species using the criterion that it must fall within, or be sister to, the species clade in a tree; Av (MRCA-tips), the mean distance between the most recent common ancestor of a putative species and its members; P (Randomly Distinct), the probability that a clade has the observed degree of distinctiveness due to random coalescent processes; Clade Support, the bootstrap or posterior probability associated with the putative species clade; and Rosenberg´s P (AB), the probability of reciprocal monophyly under a random coalescent model (for details, see Masters et al. 2011).

## ﻿Results

### ﻿Phylogenetic relationships

**Figure 1.**
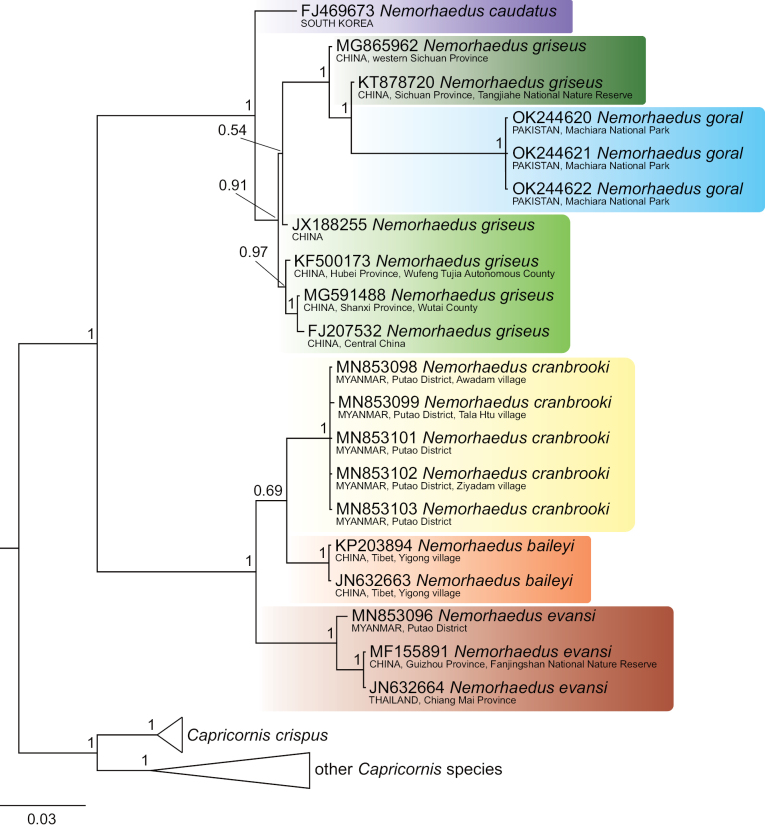
Bayesian phylogenetic tree of COI sequences of gorals and serows. Detailed tree topology is given for gorals; posterior probabilities are given at branches of interest. The scale represents substitutions per site.

**Figure 2.**
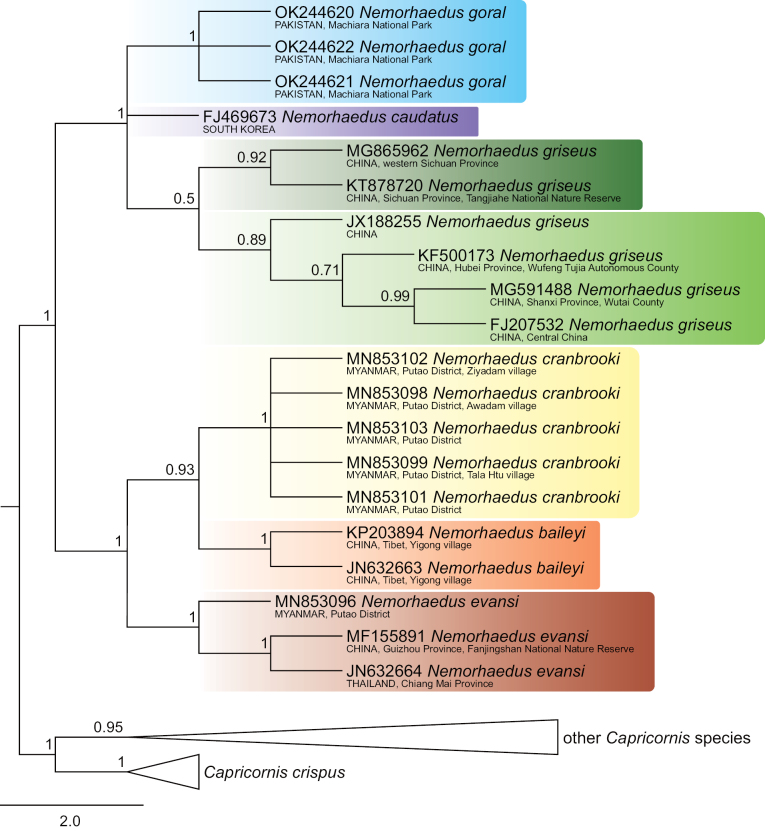
Maximum-likelihood phylogenetic tree of COI sequences of gorals and serows. Detailed tree topology is given for gorals; bootstrap values are given at branches of interest. The scale represents substitutions per site.

**Figure 3.**
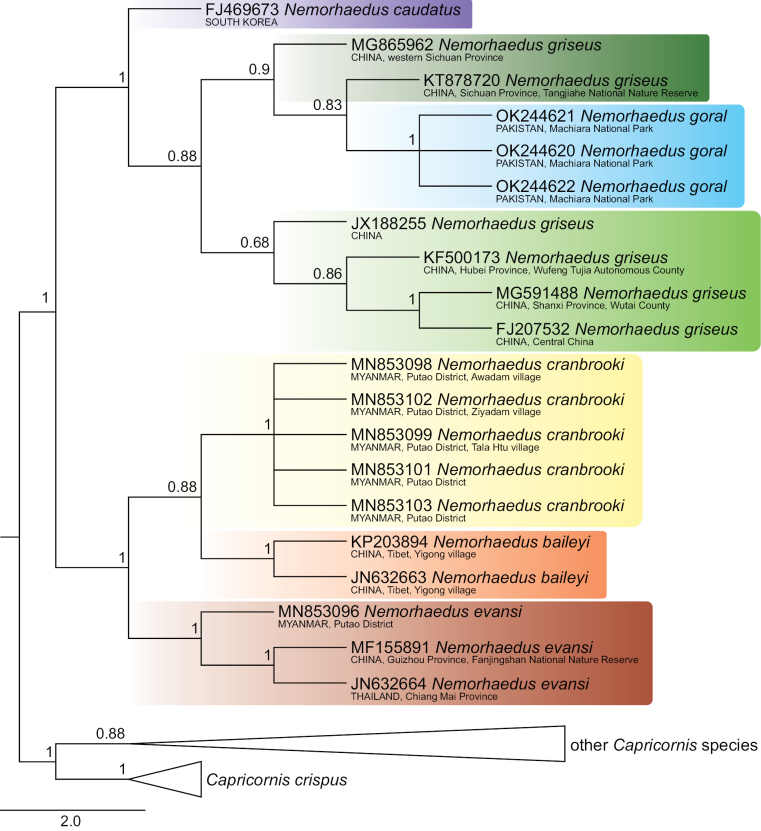
Maximum-parsimony phylogenetic tree of COI sequences of gorals and serows. Detailed tree topology is given for gorals; bootstrap values are given at branches of interest. The scale represents substitutions per site.

**Figure 4.**
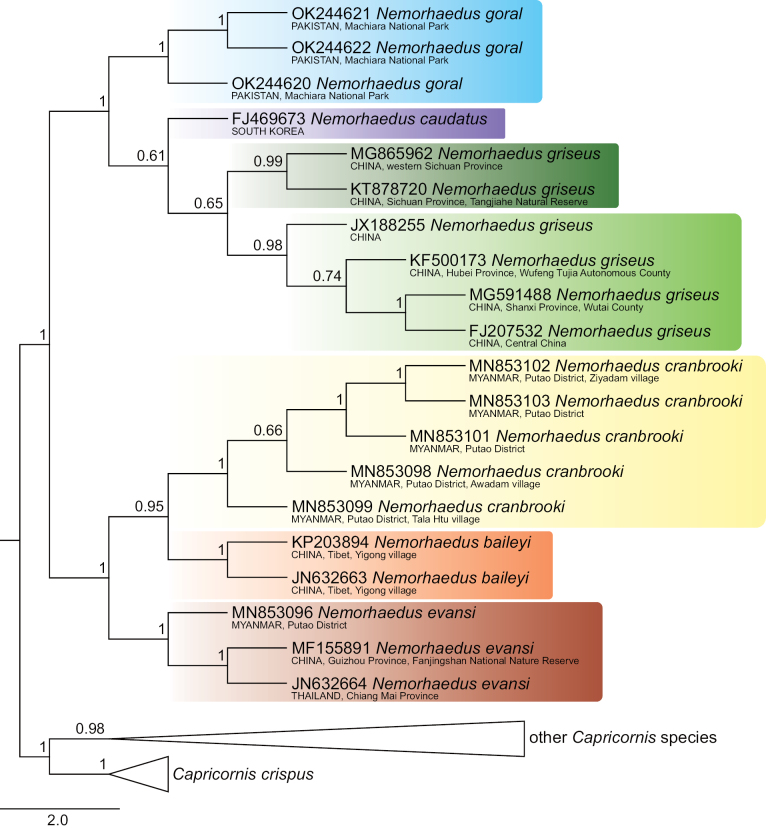
Neighbour-joining phylogenetic tree of COI sequences of gorals and serows. Detailed tree topology is given for gorals; bootstrap values are given at branches of interest. The scale represents substitutions per site.

The 134 COI sequences exhibited a significant number of variable sites, specifically 558 from all 1545 bp, of which 509 were parsimony informative. The dataset provided significant support for two goral groups, which are definable by their distribution: group I, comprising *N.baileyi*+*N.cranbrooki*+*N.evansi*, occupies the southeastern part of the goral range, whereas group II, comprising *N.goral*+*N.caudatus*+*N.griseus*, includes samples from Pakistan, Central China and South Korea (Figs 1–4).

Goral group I exhibited the topology *evansi*+(*baileyi*+*cranbrooki*) consistently in all sorts of analyses (Figs 1–4). Identical topology was obtained by Li et al. (2020), based on complete mitochondrial genomes, as well as by Joshi et al. (2022), based on partial fragments of both the cyt *b* (404 bp) and the D-loop (225 bp) genes.

Furthermore, the relationships among goral taxa in group II were dependent on an analytical approach. Specifically, the NJ and ML analyses detected *goral*+(*caudatus*+*griseus*) topology (Figs 2, 4), while the MP and BI analyses detected *caudatus*+(*griseus*–*goral*) topology, where specimens of *N.goral* formed a monophyletic cluster with the long branch nested inside *N.griseus* (Figs 1, 3). The second variant of relationships inside goral group II might be an artefact based on similarities among sequences when two specimens of *N.griseus* (KT878720, MG865962) exhibit mutual similarities, and dissimilarities to *N.goral* and the remaining *N.griseus* specimens for most nucleotide positions, but the KT878720 from Tangjiahe National Nature Reserve, Sichuan Province, China revealed a further eight (675, 693, 705, 822, 912, 963, 1029, 1482) similar positions to all *N.goral* specimens. The sequence KT878720 and the source population require further attention in relation to the quality of sequences and the possibility of the ancestral polymorphism, drift or some possible introgression event in the past. Contrary to our phylogenetic tree topology of Pakistani sequences, the samples of *N.goral* from Uttarakhand (India) form the sister group to all other goral species based on fragments of cyt *b* and the D-loop (see Joshi et al. 2022).

### ﻿Species delimitation statistics and genetic distances

The single-locus species delimitation statistics provided in their combination a support for the distinctiveness of all putative species (Tables 3, 4). Specifically, *N.baileyi*, *N.cranbrooki* and *N.evansi* from goral group I and *N.caudatus* from goral group II were well supported for both (ML and BI) gene trees, whereas *N.griseus* and *N.goral* were well supported only for the ML gene tree. As mentioned previously, the *N.griseus* sequence (KT878720) had an exceptional influence on the relationships between the *N.griseus* and *N.goral* specimens; when it was not included, *N.griseus* and *N.goral* were also well supported for the BI gene tree (data not shown).

**Table 3.** Summary statistics from the Species Delimitation plugin of Geneious for *Nemorhaedus* species recovered in the Bayesian tree using data from COI. Species are arranged alphabetically by scientific name.

Species	Closest Species	Monophyletic?	Intra	Inter - Closest	Intra/Inter	P ID (strict)	P ID (liberal)	Av (MRCA-tips)	P (Randomly Distinct)	Clade Support	Rosenberg’s P(AB)
*N.baileyi*	*N.cranbrooki*	YES	0.002	0.032	0.05	0.57 (0.42, 0.72)	0.95 (0.8, 1.0)	7.860E-4	0.05	1.00	0.02
*N.caudatus*	*N.griseus*	YES	0.00E+00	0.035	0.00E+00	0.00E+00 (0.00E+00, 0.00E+00)	0.96 (0.83, 1.00)	0.00E+00	NA	NA	0.05
*N.caudatus*	*N.griseus* (eastern subclade)	YES	0.00E+00	0.028	0.00E+00	0.00E+00 (0.00E+00, 0.00E+00)	0.96 (0.83, 1.00)	0.00E+00	NA	NA	0.1
*N.cranbrooki*	*N.baileyi*	YES	0.002	0.032	0.06	0.90 (0.77, 1.00)	0.97 (0.87, 1.0)	9.303E-4	0.05	1.00	0.02
*N.evansi*	*N.baileyi*	YES	0.010	0.063	0.16	0.69 (0.51, 0.86)	0.92 (0.77, 1.0)	0.0081	0.14	1.00	1.85E-03
*N.goral*	*N.griseus*	YES	0.002	0.077	0.02	0.78 (0.60, 0.95)	1.0 (0.85, 1.0)	7.810E-4	0.05	1.00	0.17
*N.goral*	*N.griseus* (eastern subclade)	YES	0.002	0.099	0.02	0.78 (0.61, 0.96)	1.0 (0.86, 1.0)	7.933E-4	0.05	1.00	0.01
*N.griseus*	*N.caudatus*	NO	0.019	0.035	0.54	0.57 (0.45, 0.70)	0.85 (0.75, 0.95)	0.0117	0.05	0.90	0.05

**Table 4.** Summary statistics from the Species Delimitation plugin of Geneious for *Nemorhaedus* species recovered in the maximum-likelihood tree using data from COI. Species are arranged alphabetically by scientific name.

Species	Closest Species	Monophyletic?	Intra	Inter - Closest	Intra/Inter	P ID (strict)	P ID (liberal)	Av (MRCA-tips)	P (Randomly Distinct)	Clade Support	Rosenberg’s P(AB)
*N.baileyi*	*N.cranbrooki*	YES	8.00E-6	0.025	3.2E-4	0.59 (0.44, 0.74)	0.98 (0.83, 1.0)	4.000E-6	0.05	1.00	0.02
*N.caudatus*	*N.griseus*	YES	0.00E+00	0.025	0.00E+00	0.00E+00 (0.00E+00, 0.00E+00)	0.96 (0.83, 1.0)	0.00E+00	NA	NA	0.05
*N.cranbrooki*	*N.baileyi*	YES	2.73E-4	0.025	0.01	0.93 (0.80, 1.0)	0.98 (0.88, 1.0)	1.406E-4	0.05	1.00	0.02
*N.evansi*	*N.baileyi*	YES	0.006	0.046	0.14	0.70 (0.52, 0.87)	0.93 (0.78, 1.0)	0.0056	0.81	1.00	1.85E-03
*N.goral*	*N.griseus*	YES	1.06E-5	0.078	1.3E-4	0.79 (0.62, 0.97)	1.0 (0.86, 1.0)	6.666E-6	0.05	1.00	1.85E-03
*N.griseus*	*N.caudatus*	YES	0.013	0.025	0.51	0.59 (0.47, 0.72)	0.86 (0.76, 0.96)	0.0078	0.07	0.50	0.05

Here we compare the statistics of species in much detail:

Larger values of intraspecific tree distances (see “Intra” in Tables 3, 4) indicate that the members of the species are more diverse, which applies to *N.evansi* and *N.griseus*, because they also originated from multiple distant sites. Species represented by sequences originating from a single locality/region (*goral*, *baileyi* and *cranbrooki*) have the lowest values of this metric. *N.griseus* exhibited exceptionally diverse intraspecific divergence values (nearly twice those of *N.evansi*), indicating a marked differentiation within the western subclade, consisting of samples from Sichuan Province and the eastern subclade of Hubei and Shanxi Provinces. Larger values of interspecific tree distances (see “Inter – Closest” in Tables 3, 4) indicate that the species groups are increasingly distinct. The majority of putative species (*N.baileyi*, *N.cranbrooki*, *N.griseus* and *N.caudatus*) exhibit similar values to this statistic, whereas *N.evansi* and *N.goral* have the largest values, which are two to four times higher than other putative species. Two other metrics, “P ID (strict)” and “P ID (liberal)”, show the probabilities of correctly identifying an unknown specimen of the focal species using the criteria that it must fall either within or be sister to the focal monophyletic species clade, respectively. The mean P ID (strict) were 0.6 and 0.59 as calculated with the ML and BI trees, respectively. In both analyses, *N.cranbrooki* (0.93 and 0.9) and *N.goral* (0.79 and 0.78) showed the largest value of this metric (Tables 3, 4). The mean values of P ID (liberal) were 0.95 and 0.94, as calculated with the ML and BI trees, respectively; in both analyses, all species had probabilities equal or above 0.95, with exception of *N.evansi* (0.92–0.93) and *N.griseus* (0.85–0.86). The probability that a clade has the observed degree of distinctiveness due to random coalescent processes (“P (Randomly Distinct)” in Tables 3, 4) is expressed with values between 0.05–1; all values in putative species of gorals fall within this range. All values of the probability that species will be reciprocally monophyletic under the null model of random coalescence (see “Rosenberg’s P(AB)” in Tables 3, 4) are ≤ 0.05, which support putative species as distinct species.

Uncorrected and K2P distances among goral species are shown in Table 5. Genetic divergence within the goral species ranged from 0.0 to 1.13% (Table 5). The average interspecific divergences within the two goral species groups ranged from 2.1 to 5.76%. Within goral group I, *N.baileyi* and *N.cranbrooki* showed the smallest interspecific distances, of 2.32%, whereas the largest values of interspecific distances, of 3.87%, were calculated between *N.baileyi* and *N.evansi*. In goral group II, the lowest value of interspecific distances between two geographically adjacent species, *N.griseus* and *N.caudatus*, was obtained, of 2.1%. However, the geographically most separated species in goral group II, *N.caudatus* and *N.goral*, showed the highest values of intraspecific genetic divergence. Three sequences of *N.goral* differed from the single available sample of *N.caudatus* by 5.76%. It is also important to note that high values of genetic distance, of 5.11%, also separated the remaining species in this group, *N.goral* and *N.griseus*. *N.evansi* is probably the most neglected and misidentified goral species, since it has not been considered a valid taxon by most authors (further discussed below), although it clearly represents a distinct evolutionary lineage of gorals (Hassanin et al. 2012; Li et al. 2020; Joshi et al. 2022).

**Table 5.** Matrix of genetic distances (percent sequence divergence) within and among species of genus *Nemorhaedus*. Average uncorrected (p) distances among conspecific sequences are arrayed along the diagonal, interspecific p distances are below the diagonal, and Kimura two-parameter (K2P) distances are above the diagonal. Species are arranged alphabetically by scientific name.

	*N.baileyi*	*N.caudatus*	*N.cranbrooki*	*N.evansi*	*N.goral*	*N.griseus*	*N.griseus* western group	*N.griseus* eastern group
*N.baileyi*	**0.00**	7.70	2.32	3.87	9.52	7.86	8.08	7.75
*N.caudatus*	6.73	–	7.00	6.99	5.76	2.10	3.03	1.77
*N.cranbrooki*	2.21	8.06	**0.03**	3.74	10.28	8.33	8.61	8.19
*N.evansi*	3.60	8.02	3.49	**0.60**	10.11	8.29	8.46	8.21
*N.goral*	8.09	6.43	8.62	8.52	**0.00**	5.11	4.08	5.63
*N.griseus*	6.84	2.19	7.19	7.18	4.66	**1.13**	–	–
*N.griseus* western group	7.02	2.88	7.42	7.32	3.79	–	**0.4**	1.8
*N.griseus* eastern group	6.75	1.72	7.10	7.14	5.10	–	1.58	**0.6**

## ﻿Discussion

The phylogenetic consensus on two lines of gorals – *baileyi*–*cranbrooki*–*evansi* versus *caudatus*–*goral*–*griseus* (taxa ordered alphabetically) – obtained in this study is fully concordant with some other studies based on different mt genes or the whole mitogenomes (e.g., Li et al. 2020; Joshi et al. 2022). Such a result implies that some unrelated goral taxa exhibit highly similar phenotypes (e.g., *evansi* and *griseus*), which means that their morphology could conceal their real phylogenetic relationships.

In terms of summarising various statistics relating to phylogenetic exclusivity, COI seems to support the distinctiveness of six goral taxa, specifically *N.baileyi*, *N.caudatus*, *N.cranbrooki*, *N.evansi*, *N.goral* and *N.griseus*. Our results exhibit a noticeable concordance with most attempts to revise gorals since 2005, especially with Grubb (2005), Groves and Grubb (2011) and Hrabina (2015), using morphological data, and Li et al. (2020) and Joshi et al. (2022), using mitochondrial DNA (Suppl. material 1); for exceptions, see below.

All authors of goral assessments since 2000 (Suppl. material 1) have accepted the validity of *N.baileyi*, *N.caudatus* and *N.goral*, so the morphological and genetic evidence appear to be in harmony. Agreement on the validity of *N.baileyi* and *N.caudatus* is unsurprising due to their marked phenotype, i.e., vivid orange-red colouration in *N.baileyi*, and pale grey colouration of shaggy fur and long tail tuft with white-coloured rim and dark dorsal stripe in *N.caudatus*. The acceptance of *N.caudatus* has also been supported via observations on its infertile hybrids (with *N.griseus*), under captivity conditions (Volf 1976).

### ﻿Discussion of goral taxa, in order of years when they were described

**Figure 5.**
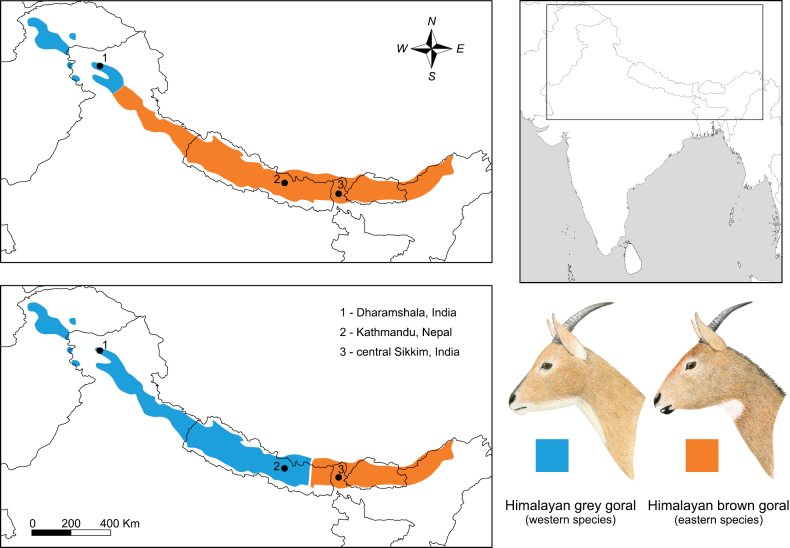
Map of distribution range of grey and brown goral following two major zoogeographical concepts with consequences of their taxonomy. The black dots represent the type localities of the three available names for Himalayan taxa: *N.bedfordi* (Lydekker, 1905) (Dharamshala, Himachal Pradesh, India), *N.goral* (Hardwicke, 1825) (Kathmandu, Nepal) and *N.hodgsoni* Pocock, 1908 (Sikkim, India). The upper map shows a scheme where the distribution boundary between the two species is formed by the Sutlej River, Himachal Pradesh, India. *N.bedfordi* here represents a valid name for the western species, and the East Himalayan species is referred to as *N.goral* (see Groves and Grubb 2011). On the contrary, the bottom map shows the situation where the border is defined east of Kathmandu. In this case, the name *N.goral* represents the name for the Western Himalayan species, while the Eastern Himalayan species is listed as *N.hodgsoni* (see Hrabina 2015).

Although the validity of *N.goral* is widely accepted because it was the first named species of the genus, it also represents the most complicated species from taxonomic and geographic perspectives. The first reason for this is that many authors still adopt the single-species concept in this genus first proposed by Allen (1930) (e.g., Chen et al. 2011; Li et al. 2012; Wu et al. 2012; Liu et al. 2017a, 2017b; Liu and Jiang 2017; Ren et al. 2018). Any studies considering multiple goral species should be aware of the geographic restriction of this group to the Himalayan region. Additionally, the grey-brown or brown-coloured gorals of the Himalayas may represent two species – *N.goral* and *N.bedfordi* (Lydekker 1905; Grubb 2005; Groves and Grubb 2011) or *N.goral* and *N.hodgsoni*, respectively (Pocock 1908; Lydekker 1913; Hayman 1961; Dolan 1963; Hrabina 2015). The two classifications differ according to the definition of the distribution boundary between both species (see Fig. 5). According to Groves and Grubb (2011), the Sutlej River in Himachal Pradesh, India forms the boundary of their distribution. In this case, *N.bedfordi* represents a valid name for the West Himalayan species, while the East Himalaya species is referred to as *N.goral* (syn. *N.hodgsoni*). In contrast, authors advocating the latter approach delimit the boundary between the two species to be approximately 100 km east of Kathmandu, Nepal. Therefore, the type locality of *N.goral* (syn. *N.bedfordi*) is contained in the distribution range of the West Himalayan species, and the name *N.hodgsoni* would thus become a valid name for the East Himalayan species, if it would be supported by species delimitation statistics in future. The sampling of the East Himalaya goral population is the major priority issue in goral taxonomy.

The second described species, *N.caudatus*, is one of the best studied goral species due to the various scientific studies from many fields based mainly on the population from South Korea. It has been consensually recognised as a valid species in all taxonomic studies adopting both morphological and molecular approaches (e.g., Groves and Grubb 2011; Hrabina 2015; Mori et al. 2019; Li et al. 2020). Although it is less susceptible to misidentification than other goral species, there exist several papers with apparent confusion, e.g., the record of observation of this species from Thingbu village, Arunachal Pradesh, India by Mishra et al. (2005). This record refers to an area approximately 3,300 km from the closest distribution ranges of *N.caudatus*, in Northeast China, Eastern Russia (Primorsky and Khabarovsk territories) and the Korean peninsula (Grubb 2005; Groves and Grubb 2011; Valdez 2011; Hrabina 2015). Most molecular studies conducted on *N.caudatus* have analysed fragments of the cyt *b* and D-loop genes and, except for one sequence from Russia, have been limited to samples from South Korea (Min et al. 2004; Jang et al. 2020). Min et al. (2004) have identified in 12 South Korean and 1 Russian sequences two haplotypes based on a short fragment of the cyt *b* gene that differed by only one nucleotide. The team also identified that the Korean and Russian sequences of *N.caudatus* are distinct from the Chinese sample of *N.caudatus* (U17861, from the San Diego Zoo). However, historical evidence (e.g., Dolan 1987; Matschei 2003) would suggest that this Chinese sequence should be reassessed as *N.griseus*. Although GenBank contains many molecular data on *N.caudatus*, the COI gene is represented by a single sample. While this does not allow for assessing intraspecific variation, the interspecific comparison with the closest related species, *N.griseus*, is notable. The average genetic distance between *N.caudatus* and *N.griseus* was calculated as 2.1% (K2P distance in Table 5), and geographically closer populations from Hubei and Shanxi Provinces of China (eastern clade of *N.griseus*) show much lower values of genetic distance to *N.caudatus* than sequences from Sichuan Province (western clade of *N.griseus*), at 1.77% and 3.03%, respectively (Table 5).

All recent morphology-based revisions have recognised the existence of *N.griseus* (Grubb 2005; Groves and Grubb 2011; Valdez 2011; Hrabina 2015), and even authors who have adopted the single-species concept list this taxon as a valid subspecies (Allen 1940; Dolan 1963; Mead 1989). However, two recent molecular-based studies have synonymised *N.griseus* with *N.goral* (Mori et al. 2019; Li et al. 2020), although this was due to misidentification of several specimens from China (Tables 1, 2). All central Chinese sequences of *N.griseus* are separated from the current study’s three Himalayan sequences of *N.goral*, from Pakistan, by a genetic distance of 5.11% (Table 5), which represents the highest value among the pair of closest species within all goral taxa. It is also important to note that in all our statistics, *N.griseus* and *N.caudatus* were assessed as the most closely related species (Tables 3–5). In contrast to all other gorals, *N.griseus* is a highly heterogeneous species with at least two clearly geographically separated maternal lineages: the western, from Sichuan Province, and the eastern, from Shanxi and Hubei Provinces. The maximum genetic distances between these two clades reach values comparable to interspecies ratios, namely 2.3% between the sequences KT878720 and FJ207532 (Suppl. material 3). Notably, these two clades seem to exhibit different diploid chromosomal numbers; specifically, a male *N.griseus* from Western Sichuan (western maternal line) showed diploid chromosomal number 2n = 54 (Shi et al. 1986; Liu et al. 1994), whereas a male and female from the eastern lineage originating from Beijing Zoo showed 2n = 56 (Soma et al. 1980; Biltueva et al. 2020; pers. com. of PH with particular holders of gorals). If these clades are further supported as distinct, they should be labelled as *N.griseus* Milne-Edwards, 1871 (western clade) and *N.henryanus* (Heude, 1890) (eastern clade).

It is also important to note that the genetic screening of COI nowhere near covers the entire distribution range of *N.griseus*. As the results of genotyping of mitochondrial fragments and microsatellite markers of nuclear DNA in animals from the northeastern limit of the species range, specifically from Songshan National Nature Reserve (Beijing) and Saihanwula National Nature Reserve (Inner Mongolia), suggest further increases in genetic distance (Yang et al. 2019), the authors have proposed separating these two populations as evolutionarily significant units that should be preserved for future generations.

While *N.evansi* was predominantly lumped with *N.griseus*-based morphological evidence, Groves and Grubb (2011), Valdez (2011) and Hrabina (2015) have argued for a morphological distinction. The first sequence, JN632664, of *N.evansi* originating in Thailand, was published by Hassanin et al. (2012), under the name *N.griseus*. The authors identified the Chinese and Thai populations of *N.griseus* as possible members of two distinct species, without taxonomic implications for the Thai voucher. Other studies analysing this sequence and other samples of this taxon have recognised it as a distinct goral species and not related to *N.griseus* at all (Li et al. 2020; Joshi et al. 2022). This implies some portion of phenotype convergence, and other examples of such phenotype convergence include *N.baileyi* and *Capricornisrubidus*, and *N.griseus* and *C.swinhoii*. Our results support this distinction based on various statistics, aligning with other genetic studies based on mtDNA (e.g., Li et al. 2020; Joshi et al. 2022). Li et al. (2020) have added three additional sequences of *N.evansi*, of which two originate from Myanmar, where the species was already documented (Valdez 2011; Hrabina 2015). However, Li et al. (2020) have also identified this species in Fanjingshan National Nature Reserve, Guizhou Province, China, which lies outside the geographical limits of the previously documented species. The presented data support the quality and distinction of the JN632664 sequence and also indicate a low mitochondrial genetic differentiation in *N.evansi* throughout almost its entire distribution range, namely Thailand, Myanmar and China. The results reveal a 0% genetic distance between the populations of Chiang Mai Province, Thailand (JN632664) and Guizhou Province, China (MF155891) (Suppl. material 3). Although the comparison is based on only two individuals, this finding is surprising given the large geographical distance (approximately 1,470 km) between these two localities. This finding seems to be partly confirmed by highly restricted haplotype and nucleotide diversity within the Thai population (Ariyaraphong et al. 2021). It may indicate that the species originated from a small founder population that has recently expanded. Since the highest value (specifically 0.4%) of intraspecific genetic distance within a single population of *N.evansi* was found by Li et al. (2020) in Putao District, Myanmar, based on a complete mitochondrion, Northern Myanmar appears to represent a candidate for such a source population. Identifying the region with the highest infraspecific variability would be helpful for effective conservation management in this taxon. Li et al. (2020) have also provided interesting insights into interspecies interactions along contact zones between *N.cranbrooki* and *N.evansi*: the voucher animal of the MN853096 sequence from Northern Myanmar is indistinguishable from *N.cranbrooki* based on pelage colouration, but has been genetically identified as *N.evansi*. Given that there are no other known red-coloured specimens of *N.evansi* from India, Myanmar, Thailand or China, and that *N.cranbrooki* is also found in the Putao District, we expect that this animal might represent a natural hybrid. In this context, it is worth mentioning that the red coat colouration appeared to be the dominant trait in two hybrid males between *N.griseus* and *N.baileyi* at Beijing Zoo (Hrabina 2015). In conclusion, *N.evansi* should be resurrected based on its genetic and morphological distinction from other goral species. Considering the relatively rich literature on the Thai and Burmese populations of *N.evansi* in recent decades, it should be remembered that they were labelled as *N.goral* via the single-species approach (Mishra et al. 1998; Chaiyarat et al. 1999), as *N.griseus* via the taxonomic concept by Grubb (2005) (Hassanin et al. 2012; Treydte et al. 2013; Buranapim et al. 2014; Khonmee et al. 2014a, b, c; Li et al. 2014; Liu and Zhang 2018; Li et al. 2019; Jangtarwan et al. 2020; Ariyaraphong et al. 2021) and as *N.caudatus* via a classification proposed by Groves and Grubb (1985) (Tougard 2001; Pintana and Lakanavichian 2013). There are also studies that have adopted more than one taxonomic scheme (Suraprasit et al. 2016; Wattanapituksakul et al. 2018; Shoocongdej and Wattanapituksakul 2020; Suraprasit et al. 2020a, 2020b; Isarankura Na Ayudhya et al. 2022).

With reference to Groves and Grubb (2011), no differences were apparent in skulls and horns between West and East Himalayan species, and their taxonomic separation is based only on pelage characteristics, which is supported by Hrabina (2015). There are no COI sequences available for *N.hodgsoni* from the eastern Himalayas, so we had to rely on the only study, by Joshi et al. (2022), that provides a comparison of another marker. Referring to these authors, two partial fragments of the cyt *b* gene of *N.hodgsoni* (MT845352, MT845353) originating from Sikkim show a genetic divergence between the West Himalayan species *N.goral* (MT845345–MT845353) that ranges from 1.2 to 3.0%. This range corresponds to the genetic divergence between *N.baileyi* and *N.cranbrooki*, for which the authors calculated values of between 2.7 and 3.0% from the same dataset.

*N.baileyi* is one of the least studied species due to its remote, geographically restricted range and the almost complete absence of comparative material in world collections. The two sequences from our analyses both have a direct link to the breeding group in the Shanghai Zoo, which is currently the only holder of this species in captivity (Shi et al. 2016; Yuan et al. 2019). The founder animals of this stock were obtained from Yigong village (30°14'N, 94°49'E), Bayi District, Tibet, China. Much knowledge of this species is based on surveys of individuals from this facility (e.g., Liu et al. 1989; Liu et al. 1994; Guo et al. 2004; Xie 2006; Xiong et al. 2013; Yuan et al. 2019). Akin to other goral species, these sequences show virtually zero genetic distance as representatives of a single locality (Suppl. material 3; Table 5).

*N.cranbrooki*, as the latest goral taxa, described in 1961 by Hayman, has been predominantly synonymised with *N.baileyi* based on morphological evidence, but the molecular data presented by Li et al. (2020) and Joshi et al. (2022) has resurrected its validity. Here, presented gene tree-based species delimitation statistics seem to support its validity as well. The estimated average value of COI genetic distances between *N.baileyi* and *N.cranbrooki*, 2.32%, is similar to the average genetic distance between *N.griseus* and *N.caudatus*, 2.10% (Table 5). The two red-coloured species were also examined karyotypically and showed differences in the number of diploid chromosomes; specifically, two males of *N.baileyi* from Tibet showed 2n = 56 (Liu et al. 1989, 1994; Huang et al. 2005), while a male *N.cranbrooki* from Myanmar showed 2n = 55 (Hsu and Benirschke 1973). The same diploid number of chromosomes in *N.cranbrooki* was documented in a female *N.evansi* from Myanmar (Wurster 1972; Hsu and Benirschke 1973). Since the current sampling of *N.baileyi* and *N.cranbrooki* is limited, a denser sampling would detect phylogenetic exclusivity with much more precision.

### ﻿Future perspectives

Considering the genetic sampling of published studies and currently inspected available GenBank and Barcode of Life Data System sequences, we recommend further attention be paid to the following taxa and populations:

1. As summarised in Suppl. material 1, our study, based on COI, did not compare populations labelled by some authors as *arnouxianus* (Zhejiang Province, China), *hodgsoni* (Sikkim, West Bengal, the western part of Arunachal Pradesh, and Bhutan) or *raddeanus* (Heilongjiang Province, China); the same absence of assessment of these taxa applies to the complete cyt *b* and the D-loop as well.
2. Considering currently restricted sampling of *N.baileyi* and *N.cranbrooki*, i.e., both species from just a single locality situated at the opposite ends of the distribution range of both taxa, we recommend that proper morphological and genetic assessments of the validity of *N.cranbrooki* and detection of distribution boundaries between both red-coloured taxa be carried out.
3. Better sampling of *N.evansi* might detect the source population for the historical expansion of this species; such a population maintaining the diversity would have exceptional conservation value. The individual associated with the sequence MN853096 should be inspected for nuclear DNA to identify its potential hybrid nature and the possible existence of hybrid zones of contact between this species and *N.cranbrooki*.
4. Consistent with our recognition of two clades within *N.griseus*, we recommend additional genetic sampling from different locations within the species range to detect their distributional boundaries (samples from Gansu and Shaanxi Provinces will play the most important role) and to recognise their genetic variability and accurate taxonomic position; from this perspective, it would also be useful to inspect the maternal genetic profile of infertile hybrid animals from Prague Zoo (see Groves and Grubb 2011, p. 254).
5. Since the sequence from the Mt. Qomolangma Nature Reserve, Tibet, China sample (JX188255, complete mitochondrion) exhibits strong genetic affinities with the eastern clade of *N.griseus* from Shanxi and Hubei Provinces, but its claimed provenance lies in the distribution range of *N.goral* sensu Hrabina 2015, based on pelage colour characteristics from this and surrounding regions as well as populations from Machiara NP and Kathmandu (e.g., figs 1, 10, 12 in Hrabina 2015), further assessment of this population is required.
6. To resolve the inconsistency in Himalayan goral taxonomy, we also recommend a genetic screening of goral populations near Kathmandu (the type locality of *N.goral*), and a subsequent comparison with samples from further western and eastern localities.
7. Since some taxa also seem to be supported by different chromosomal numbers, it would be worth inspecting the chromosomal variability of more specimens per goral taxa.
8. For species that are represented by samples from a single locality (i.e., *N.goral*, *N.baileyi*, *N.cranbrooki* and *N.caudatus*), we recommend a broader sampling across the entire distribution area to understand the true intraspecific diversity and phylogenetic position of each species.

In summary, COI exhibited a good resolution for separation species, but it did not contain a strong resolving power in the case of phylogenetic relationships. Since it provides very similar results to those of other mitochondrial genes (cyt *b* and the D-loop) used in assessments of goral phylogeny and taxonomy, we recommend sequencing of the complete sequence of all three mt genes for degraded samples from collections and the field. If the sample is well preserved, sequencing of the complete mitochondrial genome is highly recommended. It is worth mentioning that gorals are believed to represent sedentary caprines, with a limited male-based dispersal (Heptner et al. 1988); all results based on mt data might therefore provide a better structure in results than results based on Y-chromosome, microsatellite and genomic data. Therefore, multilocus or genomic data are highly recommended for the deciphering of all the above-mentioned issues, such as the detection of real phylogenetic relationships, the timing of diversification, and the portion of convergence and interspecific gene flows that could serve as sources of adaptive variation (Jónsson et al. 2014; Figueiró et al. 2017; Rakotoarivelo et al. 2019).

Considering complicating factors in deciphering goral phylogeny and taxonomy (see Introduction), we fully agree with Groves (2006) and Gutiérrez et al. (2017), who recognise the necessity to associate as much associated data with voucher specimens as possible. In practice, we highly recommend including a photograph of the DNA voucher specimens and detailed information about geographic origin in supplementary materials of published studies.

Incidentally, authors should be aware that excessive lumping of threatened taxa may have disastrous consequences for the future conservation prospects of forgotten narrow endemic ungulate taxa (Gippoliti et al. 2018). The deciphering of the goral taxonomy would represent a useful objective platform for rational conservation actions, reducing the negative human impact on this unique group of caprines.

## ﻿Additional information

### Author ORCIDs

Petr Hrabina https://orcid.org/0009-0002-3159-3737

Ludmila Pernerová https://orcid.org/0009-0004-3628-4647

Josef Suchomel https://orcid.org/0000-0002-6455-135X

Jan Robovský https://orcid.org/0000-0001-8720-9314

### Data availability

All of the data that support the findings of this study are available in the main text or Supplementary Information.

## Supplementary materials

Supplementary material 1 Goral species recognized in the 20^th^ and 21^st^ centuries across several basic sources, which seemed to assess gorals independently and/or using different data Data type xlsx Explanation note Taxa are arranged alphabetically by scientific names. https://binary.pensoft.net/file/915290This dataset is made available under the Open Database License (http://opendatacommons.org/licenses/odbl/1.0/). The Open Database License (ODbL) is a license agreement intended to allow users to freely share, modify, and use this Dataset while maintaining this same freedom for others, provided that the original source and author(s) are credited.Petr Hrabina, Ludmila Pernerová, Josef Suchomel, Jan Robovský

Supplementary material 2 List of taxa and GenBank accession numbers used for the original gene analysis with 134 COI sequences Data type xlsx Explanation note Taxa are arranged alphabetically by scientific names. https://binary.pensoft.net/file/915291This dataset is made available under the Open Database License (http://opendatacommons.org/licenses/odbl/1.0/). The Open Database License (ODbL) is a license agreement intended to allow users to freely share, modify, and use this Dataset while maintaining this same freedom for others, provided that the original source and author(s) are credited.Petr Hrabina, Ludmila Pernerová, Josef Suchomel, Jan Robovský

Supplementary material 3 Genetic Kimura two-parameter distances for COI genes among *Nemorhaedus* and *Capricornis* species Data type xls https://binary.pensoft.net/file/915292This dataset is made available under the Open Database License (http://opendatacommons.org/licenses/odbl/1.0/). The Open Database License (ODbL) is a license agreement intended to allow users to freely share, modify, and use this Dataset while maintaining this same freedom for others, provided that the original source and author(s) are credited.Petr Hrabina, Ludmila Pernerová, Josef Suchomel, Jan Robovský
